# Comparison of Effectiveness Between Providence Nighttime Versus Full-Time Brace in Adolescent Idiopathic Scoliosis: A Narrative Review

**DOI:** 10.3390/medsci14010036

**Published:** 2026-01-09

**Authors:** Ana Belén Jiménez-Jiménez, Elena Goicoechea-Rey, Pablo Padial López-Durán, Alicia María Rodríguez-Mármol, María Nieves Muñoz-Alcaraz, Fernando Jesús Mayordomo-Riera

**Affiliations:** 1 Interlevel Clinical Management Unit of Physical Medicine and Rehabilitation, Reina Sofía University Hospital, Córdoba and Guadalquivir Health District, 14011 Córdoba, Spain; anab.jimenez.jimenez.sspa@juntadeandalucia.es (A.B.J.-J.); pablo.padial.sspa@juntadeandalucia.es (P.P.L.-D.); aliciam.rodriguez.marmol.sspa@juntadeandalucia.es (A.M.R.-M.); fernandoj.mayordomo.sspa@juntadeandalucia.es (F.J.M.-R.); 2 Maimónides Biomedical Research Institute of Cordoba (IMIBIC), Reina Sofia University Hospital, University of Córdoba, 14004 Córdoba, Spain; 3 Department of de Applied Physics, Radiology, and Physical Medicine, University of Córdoba, 14004 Córdoba, Spain; 4 Faculty of Medicine and Nursing, University of Córdoba, 14004 Córdoba, Spain; h92goree@uco.es

**Keywords:** adolescent idiopathic scoliosis, providence nighttime brace, full-time brace, therapeutic adherence, health-related quality of life and psychosocial impact

## Abstract

**Background/Objectives**: Adolescent idiopathic scoliosis (AIS) is a three-dimensional deformity of the spine with multifactorial etiology. Its treatment is conservative and/or surgical. The most commonly used conservative method is a full-time brace. However, nighttime braces have recently gained prominence, offering improved tolerance and a positive impact on health-related quality of life. The main objective of this study was to conduct a narrative review of published articles comparing the effectiveness of Providence nighttime versus full-time brace use to determine whether nighttime use is an effective option for improving therapeutic adherence, health-related quality of life, and psychosocial impact. **Methods**: A scientific literature search was conducted using the Scopus and PubMed databases. We searched for randomized controlled trials (RCTs), meta-analyses, systematic reviews and retrospective comparative studies reported in English from 2019 to 2024. The literature search was conducted from October to April 2024. Different combinations of the terms and MeSH terms “adolescent”, “idiopathic”, “scoliosis”, “Providence”, “full-time” and “brace” connected with various Boolean operators were included. **Results**: Overall, 70 articles were reviewed from the selected database. After removing duplicated papers and title/abstract screening, 10 studies were included in our review. The results showed that nighttime brace use has similar results in terms of effectiveness to full-time brace use in mild to moderate curves. However, nighttime brace use improves therapeutic adherence, health-related quality of life and psychosocial impact. Nevertheless, the effectiveness of night braces depends on factors such as curve type, magnitude, and bone maturity. So, in patients with moderate-severe curves and high growth velocity, it is important to reconsider the type of brace, as in these cases night braces alone may be ineffective in slowing the progression of the curve. **Conclusions**: Providence nighttime brace could be an effective treatment and better tolerated alternative to full-time brace in specific cases. This approach could improve therapeutic adherence. Nevertheless, more controlled and homogeneous studies are needed to establish definitive recommendations.

## 1. Introduction

Adolescent idiopathic scoliosis (AIS) is a common clinical condition that affects the spine and is characterized by the appearance of a three-dimensional deformity in the absence of vertebral abnormalities or associated syndromes that would justify it. It affects 1–3% of the general population, predominantly women, and can develop during the perinatal period, early childhood, or adolescence [1,2]. The etiopathogenesis is multifactorial [3]. In this sense, various hypotheses have been proposed regarding its origin. These include growth disorders, intervertebral disc abnormalities, central nervous system dysfunctions, and muscle imbalances, among others [4]. In recent decades, several articles published in the literature have pointed to the relevance of genetic etiology in this clinical entity. In fact, certain genetic markers that may be involved in the development of this pathology and a greater presence of these genes is related to greater severity and possibility of progression of the curves [5].

**Figure 1:**
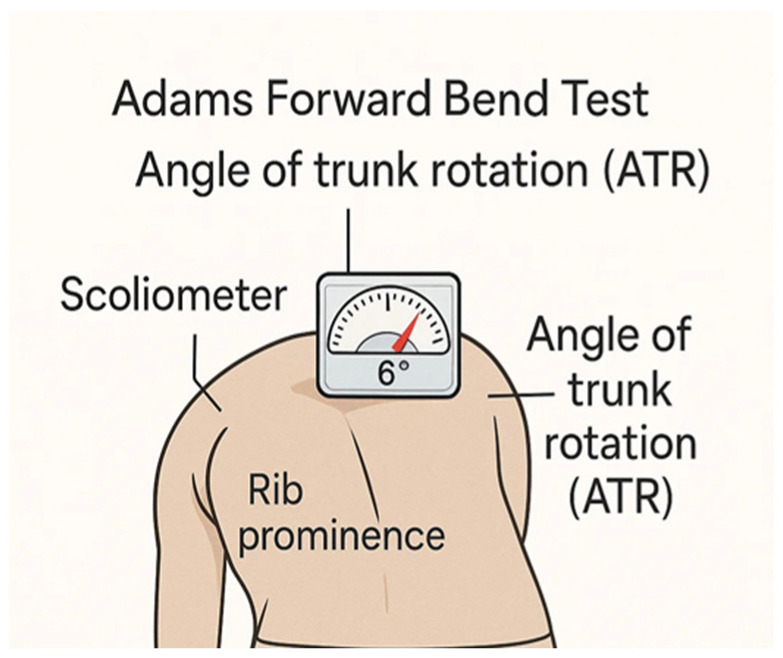
Adams test and ATR measurement with Scoliometer.

In its diagnostic approach, the screening test most frequently used in daily clinical practice is the Adams test. This consists of the patient bending their torso forward so that we can then stand behind them, level with their shoulder blades, and observe whether there is any asymmetry in the torso or hump. The test is positive when a hump appears at the dorsal or lumbar level, indicating possible vertebral rotation. In addition, to improve the performance of this test, a scoliometer can be used (Figure 1). This is an instrument that measures the angle of trunk rotation (ATR) with the patient leaning forward in the same position as previously. According to the results, an ATR of less than 5° is considered a scoliotic attitude and is not clinically relevant; an ATR between 5° and 9° should be reevaluated after six months; and finally, if the ATR is greater than or equal to 10°, standing full-spine radiograph is recommended to confirm possible scoliosis [6].

**Figure 2:**
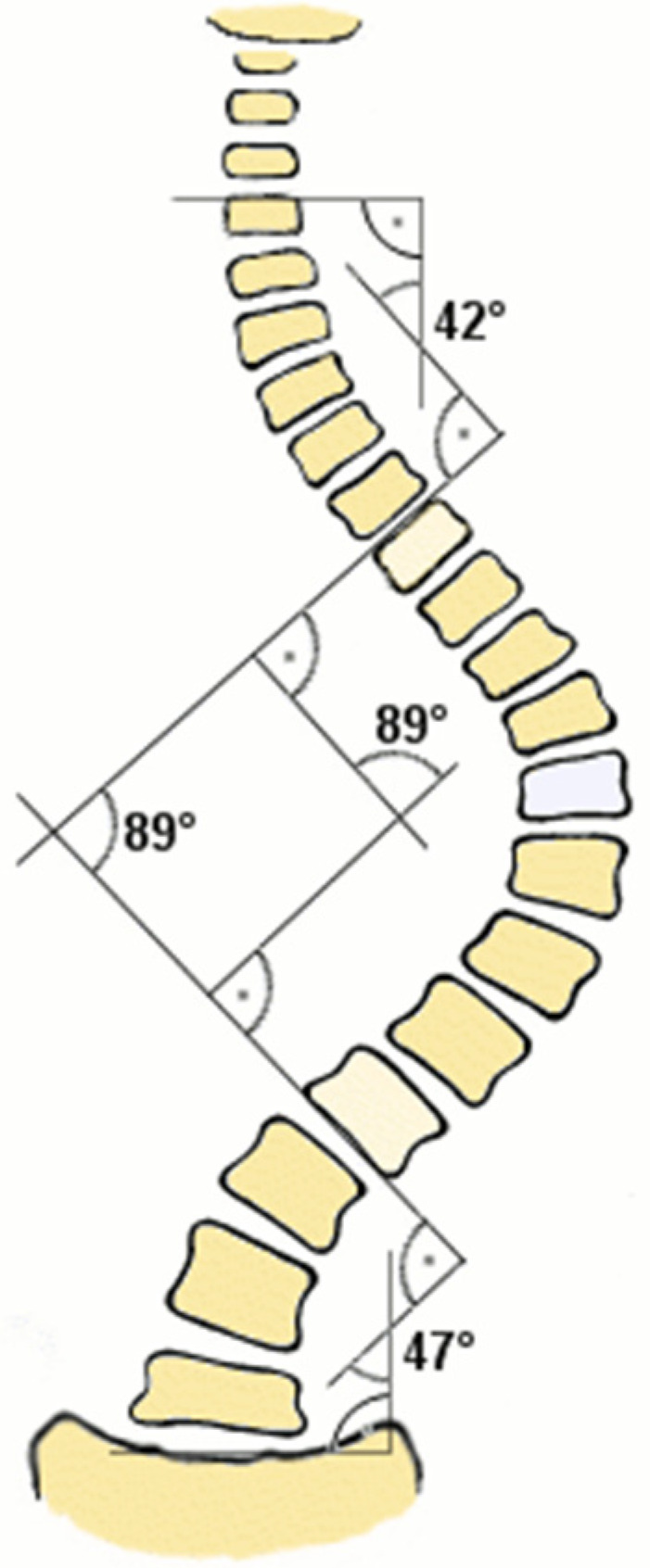
Diagram of Cobb angle measurement to assess the magnitude of the curve [9].

In this regard, a standing full-spine radiograph is usually requested. In this EOS X-ray standing, scoliosis is shown as a three-dimensional deformity of the spine with the following characteristics: presence of a frontal curvature of at least 10°, a decrease, or even an inversion, of the sagittal curvatures, and axial rotation. In this regard, these projections allow doing a correct differential diagnosis; assessing the magnitude, type of curve, and maturity of the axial skeleton using the Risser stage [1]. Moreover, in standing full-spine radiograph, the magnitude of the curve is calculated using the Cobb method, which is based on measuring the Cobb angle [7]. The Cobb angle (Figure 2) is the angle formed by the most inclined vertebrae at the top and bottom of the curve. A line is drawn perpendicular to the upper plate of the upper limiting vertebra and another line perpendicular to the lower plate of the lower limiting vertebra, and where both lines intersect, the Cobb angle is obtained [6]. The Scoliosis Research Society (SRS) suggests that a diagnosis of scoliosis would be confirmed with a Cobb angle of 10° or greater [8].

**Figure 3:**
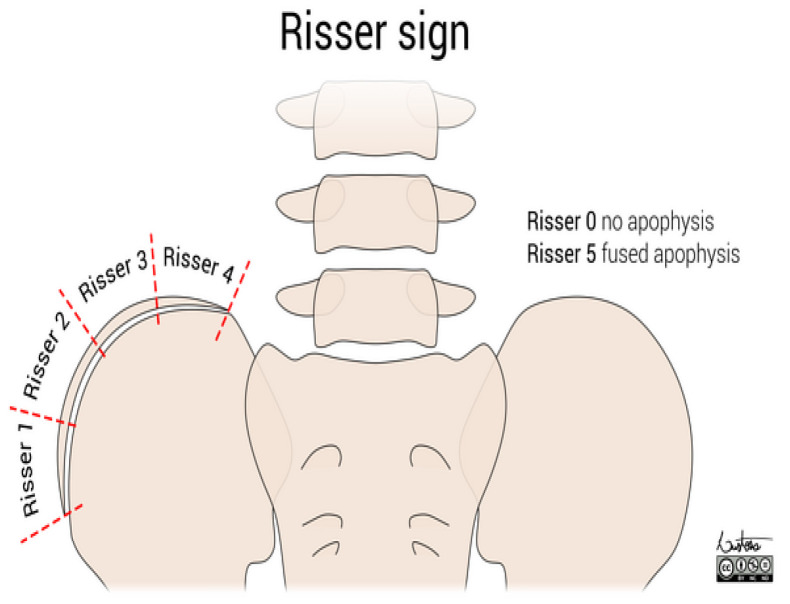
The iliac crest divided according to the stages of the Risser scale [10].

Besides, X-rays can also be used to assess skeletal maturity using the Risser stage. As bone maturity progresses, ossification of the growth cartilage develops in the iliac crest from the anterior superior iliac spine to the posterior superior iliac spine, allowing the crest to be divided into quarters that correspond to stages of maturity, from stage 0 to stage 5 (Figure 3). Stage 0 corresponds to an absence of ossification. Stage 1 would be in the first quarter, followed by stage 2, stage 3, and finally stage 4. When maturation is complete, Risser stage 5 is reached, in which the pelvis is completely ossified and the maturation line is no longer visible [9].

On the other hand, the progression of the curve is an important prognostic factor in AIS. Progression is considered to exist when there is an increase of more than 5° in the Cobb angle compared to the last measurement or when it is above 45° at follow-up. This progression depends on multiple factors, such as the location of the curve, sex, age, degree of skeletal maturity, and initial magnitude of the curve, among others. In this regard, a younger patient and a lower bone maturity are associated to a greater risk of curve progression and, consequently, greater structuring and a worse prognosis [10].

In relation to the therapeutic approach to this condition, treatment options are individualized, taking into account various factors: the type and degree of the curve, the patient’s age, and the number of years of growth remaining until skeletal maturity, among others. In respect to treatment, this may initially be conservative with the braces, although in certain cases the rapid progression of the curve necessitates surgical treatment. Therefore, brace treatment is indicated mainly for moderate curves with a Cobb angle of 25–40° and a high risk of progression. Although the use of braces has shown to be effective, its success will depend on adherence to treatment, design, device fit, and psychological support. The main objective of conservative treatment is not to correct the deformity of the spine, but to slow the progression of the curve and, secondarily, to prevent aesthetic and functional repercussions [11].

In respect of braces, the most commonly used today are full-time braces such as the Boston brace (specially designed for moderate curves and thoracolumbar or lumbar curves) or the Cheneau brace (specially designed for complex thoracic and thoracolumbar curves). However, its effectiveness varies depending on patient characteristics (type of curve, skeletal maturity, and initial response to the brace) [12].

Nevertheless, in recent years, the use of night braces has become more important as they have less psychosocial impact on patients and lead to greater adherence to treatment. Specifically, one type of night brace is the Providence, a rigid brace that applies controlled corrective forces—direct, lateral, and rotational—to the spine to align it with the central axis and slow the progression of the curve [13]. Considering the above, serial X-rays are performed to monitor the progression of the curve, generally every 6 months, as indicated by the International Society on Scoliosis Orthopedic and Rehabilitation Treatment (SOSORT) consensus committee. Thus, an increase of 5° or more between two X-ray checks would be considered progression of the curve [14].

It is well-known from studies comparing Providence nighttime braces and full-time braces for AIS that they might bring mixed results, with some indicating they are equally effective while others suggest full-time braces may be more effective in certain cases. Some research suggests that Providence nighttime braces may offer better in-brace correction and higher patient compliance; however, the final long-term success rates are similar to those of full-time braces. Factors such as patient age, sex, and curve characteristics significantly influence the outcomes of both treatment types. For this reason, the main objective of this review was to conduct a narrative review of published articles comparing the effectiveness of Providence nighttime versus full-time brace use to determine whether nighttime use is an effective option for improving therapeutic adherence, health-related quality of life, and psychosocial impact.

In addition, it is important to take into account that the progression of the curve can affect not only spinal morphology, but also lung function, postural balance, and, in many cases, self-image perception, with significant psychological repercussions. For this reason, early and effective diagnostic and therapeutic intervention is crucial to achieving a better long-term prognosis. In most cases, the long-term prognosis is favorable.

## 2. Materials and Methods

A comprehensive review was conducted using the Scopus and PubMed databases. The literature search was conducted from October to April 2024. We applied following inclusion criteria to ensure the relevance, quality, and consistency of the studies: (1) studies published between 2019 to 2024; (2) especially, studies with greater scientific evidence (randomized controlled trials (RCTs), meta-analyses, systematic reviews, retrospective comparative studies), but also, studies with lower evidence as cases series; (3) studies focus on the treatment with the Providence brace versus full-time brace wear in patients with AIS; (4) articles published in English; and (5) studies with full text access.

Different combinations of the terms and MeSH terms “adolescent”, “idiopathic”, “scoliosis”, “Providence”, “full-time” and “brace” connected with various Boolean operators were included.

The exclusion criteria were as follows: (1) studies published outside the range of 2019 to 2024; (2) studies not focused on treatment with the Providence brace versus full-time brace wear in patients with AIS; (3) articles published in a language other than English; and (4) studies without full text access.

The following data were extracted: (1) title; (2) authors; (3) publication year; (4) design; (5) characteristics of study participants (therapeutic adherence, health-related quality of life, and psychosocial impact); (6) intervention and (7) outcomes.

To conduct this narrative review of the literature and a qualitative analysis of the studies obtained, we followed the recommendations of the SANRA guidelines, and their six quality criteria (justify the topic; state the objectives; describe the literature; present the evidence found; provide adequate discussion; and highlight the relevance of the topic).

## 3. Results

The initial bibliographic search was in the Scopus and PubMed databases using the criteria mentioned and it showed a total of 70 records. Of these studies, 39 articles were excluded due to temporal and methodological criteria: 32 because they were not within the established date range (2019–2024) and 7 articles for not meeting the minimum scientific evidence criteria.

After this initial screening, 31 studies were considered potentially relevant. However, 21 additional articles were excluded because these did not take into account variables of interest. A final selection of 10 articles that met all the inclusion criteria established was included in this narrative review.

Table 1 presents the data from these studies, classified according to author and year of publication.

## 4. Discussion

The objective of this study was to conduct a narrative review of the articles available in the scientific literature comparing the effectiveness of the Providence night brace and the full-time brace in the treatment of AIS.

Traditionally, full-time bracing has been the mainstay of conservative treatment in patients with AIS with low bone maturity and/or moderate spinal curvature. However, in certain cases, the use of a brace for 24 h is associated with lower therapeutic adherence and less satisfactory results in the correction of spinal deformity. For this reason, new therapeutic strategies that increase adherence, such as the Providence night brace, have been developed in recent decades.

Several studies compare the use of night braces with other 24-h braces to assess their effectiveness in terms of curve progression in mild-moderate curves and Risser stage ≤ 2.

Capek, V., et al. [21], Simony, A., et al. [17], and Davis, L., et al. [18] showed high success rates with the Providence night brace, which may have comparable efficacy to full-time brace in moderate, lumbar, or thoracolumbar curves. Additionally, in patients with greater skeletal maturity and/or curves of less than 30°, the effectiveness was higher due to greater adherence to the nighttime regimen. This suggests that, in carefully selected patients, the night brace could be a useful and effective therapeutic option.

In the same way, in the review by Buyuk, A. F., et al. [20], similar curve progression rates were found, supporting the previously described idea that night braces could be as effective as 24-h braces in thoracolumbar or lumbar curves and Risser stages ≤ 2. However, the conclusions are limited by the sample size and quality of the studies included.

On the other hand, other studies, such as the systematic review by Ruffilli, A., et al. [19], and Karimi, M. T., et al. [16], found no statistically significant differences when comparing the progression of the curve in both braces. Nevertheless, in the subgroup with a Cobb angle of 25–35°, the Providence brace was better at preventing curve progression > 5° (*p* = 0.017). Furthermore, in narrative reviews that include several studies, it is difficult to find a clinically useful “mean Cobb angle” because the heterogeneity of the studies prevents meaningful grouping of these data.

In contrast to the above, in patients with thoracic curves, double curves, or curves greater than 35°, several articles indicate that full-time brace use is insufficient to slow curve progression, whereas night braces are sufficient. Capek, V., et al. [21,22], found statistically significant differences (*p* = 0.007) in the success rate in favor of full-time braces versus night braces (59% vs. 46%, respectively). However, subgroup analyses showed that night braces were effective in slowing the progression of curves in cases of curves less than 30°, lumbar curves, and postmenarcheal patients.

There are certain discrepancies between the articles regarding the type of brace recommended for thoracic curves. In this regard, the retrospective study by Ohrt-Nissen et al. [15] in patients with thoracic curves showed that the Providence brace was associated with greater in-brace correction (68% Providence vs. 30% Boston, *p* < 0.001). Therefore, the Providence brace could be a feasible therapeutic option even in thoracic curves in specific patients. Regarding the progression of thoracic curves, this study found that the success rate was similar for night braces and full-time braces (40% vs. 35%, respectively), although not statistically significant (*p* = 0.838). Therefore, further studies are needed since the degree of initial curve deviation was the only predictor of treatment failure (*p* = 0.024), with neither the type of brace nor the age of the patient at the start of treatment being decisive factors.

In addition to the clinical efficacy of the night brace, adherence to this treatment is particularly important to consider. Studies such as those by Capek, V., et al. [21] and Karimi, M. T., et al. [16] indicated high therapeutic adherence to the Providence brace, possibly due to the shorter daily wear time.

Another noteworthy aspect considered in this narrative review is the biomechanical impact of the type of brace on the correction of spinal deformity in the sagittal profile. In this regard, studies such as those by Heegaard, M., et al. [23] demonstrated that the Providence brace better preserved physiological thoracic kyphosis, while the full-time brace tended to produce hypokyphosis. This finding is clinically relevant, as this alteration could compromise the overall biomechanics of the spine in the long term. On the hand, in this article, no differences were observed between night braces and full-time braces in terms of slowing down the progression of the curve, as factors such as curve severity, Risser stage, and adherence to treatment influence the outcome. In patients with moderate-severe curves and faster growth rates, the effectiveness of the night brace on the curve may be less.

It should be noted that some of the studies analyzed had a number of limitations related to poorly defined selection criteria, low methodological quality, heterogeneity in sample groups, or insufficient sample sizes. This makes it difficult to draw concise conclusions and extrapolate them to daily practice. In this regard, the systematic review by Kuru Çolak, T., et al. [24], reflected a high degree of heterogeneity in the methodology of the studies it included on night braces.

In addition, most of the studies included were retrospective and some had small sample sizes, no control of therapeutic compliance, no control group, or variability in inclusion criteria and follow-up. Many of these studies did not obtain sufficient evidence to determine that the night brace was equivalent in effectiveness to the full-time brace [24].

Despite these limitations, a study known as the “Bracing Adolescent Idiopathic Scoliosis” (BASIS) study is currently underway. It is a randomized multicenter clinical trial in the United Kingdom whose main objective is to determine whether night braces are non-inferior to full-time braces in preventing treatment failure. It includes 780 patients between the ages of 10 and 15 with curves between 20° and 40° and is designed considering psychological and quality of life evaluation criteria. Although the results are not yet available, we mention it because of its relevance as the first research of higher methodological quality from which more accurate conclusions can be drawn about the effectiveness of night braces [25].

## 5. Conclusions

Following this narrative review, we support the idea that the Providence brace could be an effective therapeutic alternative to full-time bracing for adolescent idiopathic scoliosis in selected patients. Although better designed, higher quality studies are needed, the review supports that it is a possible recommendation in thoracolumbar or lumbar curves, Risser ≥ 2, in post-menarcheal females, in patients with a tendency to hypokyphosis or flat sagittal profile, and in patients who are expected to have poor adherence to full-time bracing. In these patients, nighttime brace improves therapeutic adherence, health-related quality of life and psychosocial impact. On the other hand, it is important to note that the effectiveness of night braces depends on factors such as curve type, magnitude, and bone maturity. In patients with moderate-severe curves and high growth velocity, it is important to reconsider the type of brace, as in these cases night braces alone may be ineffective in slowing the progression of the curve.

As previously indicated, this narrative review highlights the need for more studies with a more rigorous methodology to better define the characteristics and/or clinical conditions of patients who could benefit each type from of brace. This will make it possible to establish with certainty the non-inferiority of night braces compared to full-time braces, not only in terms of slowing the progression of the curve, but also in terms of therapeutic adherence and psychosocial impact.

## Figures and Tables

**Table 1:** Results of selected studies in Narrative Review.

Study	Objective	Methods	Conclusion
Ohrt-Nissen, S., et al., 2019 [15]	To compare the effectiveness of the Providence night brace with the Boston 18-h brace on thoracic curves in AIS.	Retrospective cohorts (N: 77). Level of evidence III. Cobb angle 25–40° (T7–T11), Risser ≤ 2. Average age: 12 years old. Variables: Cobb angle. Curve progression and in-brace correction were evaluated. Follow-up: 2 years.	The Providence brace had greater in-brace correction (68% Providence vs. 30% Boston, *p* < 0.001). There were no significant differences in curve progression (*p* > 0.05).
Karimi, M. T., et al., 2019 [16]	To determine the efficacy and effectiveness of using night splints versus full-time splints in AIS.	Literature review (19 articles). Level of evidence V. Cobb angle 20–40°, Risser ≤ 2. Average age: 12–14 years old. Variables: Cobb angle. Curve progression and therapeutic adherence were evaluated. Follow-up 2–3 years.	The Providence night brace was more effective and efficient in therapeutic adherence, emotional state, and avoiding surgery in curves < 35° (*p* < 0.05). It is difficult to find a “mean Cobb angle” because the heterogeneity of the studies.
Simony, A., et al., 2019 [17]	To evaluate the effectiveness of the Providence 8-h night brace.	Prospective cohort studies (N: 124). Level of evidence III. Cobb angle 20–45°. Average age: 13 years old. Variables: Cobb angle. Curve progression was evaluated. Follow-up 2–3 years.	The Providence brace demonstrated an overall success rate of 89% in preventing curve progression, comparable to that reported for 24-h braces (*p* < 0.05).
Davis, L., et al., 2019 [18]	To determine the effectiveness of the Providence night brace in AIS.	Case series study (N: 56). Level of evidence V. Cobb angle 25–40°, Risser ≤ 2. Average age: 12 years old. Variables: Cobb angle. Curve progression was evaluated. Follow-up 2–3 years.	The Providence brace was effective in slowing the progression of the curve in patients with Risser ≥ 1 and curves with the apex at T10 or below (*p* < 0.05).
Ruffilli, A., et al., 2021 [19]	To evaluate the effectiveness of nighttime brace use compared to traditional thoracolumbar sacral braces in AIS.	Systematic review (7 articles, N: 400). Level of evidence I. Cobb angle 25–40°, Risser ≤ 2. Average age: 10–18 years old. Variables: Cobb angle. Curve progression and in-brace correction were evaluated. Follow-up 2–3 years.	The Providence brace had greater in-brace correction. But, no differences were found in curve progression. In subgroup with Cobb angle of 25–35°, the Providence brace was better at preventing curve progression > 5° (*p* = 0.017).
Buyuk, A. F., et al., 2022 [20]	To study the effectiveness of nighttime braces as an alternative therapy to full-time AIS braces.	Systematic review and meta-analysis (9 studies, N: 595). Level of evidence I. Cobb angle 25–40°, Risser ≤ 2. Average age: 9–17 years old. Variables: Cobb angle. Curve progression was evaluated. Follow-up 2–3 years.	Night braces showed similar effectiveness to full-time braces in thoracolumbar/lumbar curves, Risser ≤ 2, (*p* > 0.05). The conclusions are limited by sample size and quality of studies.
Capek, V., et al., 2022 [21]	To compare the effectiveness of the Providence night brace versus the Boston full-time brace in AIS.	RCT (N: 111). Level of evidence I. Cobb angle 20–40°, Risser ≤ 2. Average age: 13 years old. Variables: Cobb angle, therapeutic adherence, skeletal maturity. Curve progression, in-brace correction and therapeutic adherence were evaluated. Follow-up 2–3 years.	The Providence brace had greater in-brace correction. But, no differences were found in curve progression. Adherence to treatment was significantly higher with the Providence night brace (*p* = 0.017).
Capek, V., et al., 2023 [22]	To compare the effectiveness of Providence night splints versus full-time Boston splints in AIS.	Retrospective cohort study (N: 358). Level of evidence III. Cobb angle 20–40°, Risser ≤ 3. Average age: 13 years old. Groups: Providence brace and Boston brace Variables: Cobb angle, Risser stages, therapeutic adherence. Curve progression and therapeutic adherence were evaluated. Follow-up 2–3 years.	The Boston brace was effective at preventing curve progression in prepubertal patients or thoracic curves > 30° (*p* = 0.029). The Providence brace showed greater in-brace correction and adherence for lumbar and mild-moderate curves.
Heegaard, M., et al., 2024 [23]	To evaluate the impact of the Providence brace on the sagittal profile in AIS and compare it with brace 24 h a day.	Retrospective cohort study (N: 124). Level of evidence III. Cobb angle 20–45°, Risser < 4. Average age: 9–17 years. Variables: Cobb angle, sagittal vertical axis. The degree of kyphosis and curve progression were evaluated. Follow-up 2–3 years.	The night brace preserves kyphosis and presents a lower risk of developing flat back deformity. Curve progression of >50° was greater in the night brace than in the full-time brace (*p* = 0.028).
Kuru Çolak, T., et al., 2024 [24]	To review and analyze the literature to determine whether night braces are effective in AIS.	Systematic review (22 articles). Level of evidence I. Cobb angle 25–40°, Risser ≤ 2. Average age: 9–17 years old. Variables: Cobb angle, surgery rate. Curve progression, surgery rates, quality of life, and psychosocial impact were evaluated. Follow-up 2–3 years.	There is insufficient evidence to support the use of night braces in the treatment of AIS due to limitations on the low quality of existing studies.

RCT: Randomized controlled trial; AIS: Adolescent Idiopathic Scoliosis.

## Data Availability

No new data were created or analyzed in this study.

## Abbreviations

The following abbreviations are used in this manuscript:

AIS	Adolescent Idiopathic Scoliosis
ATR	Angle of Trunk Rotation
RCT	Randomized Controlled Trial
SRS	Scoliosis Research Society
SOSORT	International Society on Scoliosis Orthopedic and Rehabilitation Treatment
